# Regulation of calcitonin gene-related peptide expression through the COX-2/mPGES-1/PGE2 pathway in the infrapatellar fat pad in knee osteoarthritis

**DOI:** 10.1186/s12944-018-0864-8

**Published:** 2018-09-11

**Authors:** Jun Aikawa, Kentaro Uchida, Shotaro Takano, Gen Inoue, Dai Iwase, Masayuki Miyagi, Manabu Mukai, Shintaro Shoji, Hiroyuki Sekiguchi, Masashi Takaso

**Affiliations:** 1 Department of Orthopedic Surgery, Kitasato University School of Medicine, 1-15-1 Minami-ku Kitasato, Sagamihara City, Kanagawa 252-0374 Japan; 2 Shonan University of Medical Sciences Research Institute, Nishikubo 500, Chigasaki City, Kanagawa 253-0083 Japan

**Keywords:** Calcitonin gene-related peptide/SE, Osteoarthritis, knee, Adipose tissue/PP, Prostaglandin E2

## Abstract

**Background:**

The infrapatellar fat pad (IFP) is implicated in knee osteoarthritis (KOA). Calcitonin gene-related peptide (CGRP), a vasoactive neuropeptide expressed in joint tissues and synovial tissues (ST), was recently found to be associated with KOA progression and pain. CGRP is expressed in the IFPs of human KOA patients; however, its regulation has not been elucidated.

**Methods:**

IFPs and STs were harvested from 138 KOA patients during total knee replacement (TKR) and analyzed for CGRP, cycloxygenase-2 (COX-2), and microsomal prostaglandin E synthase-1 (mPGES-1) expression using real-time polymerase chain reaction (PCR). To investigate CGRP regulation by prostaglandin E2 (PGE2), adipocytes (Ad) and the stromal vascular fraction (SVF) were harvested from IFPs using collagenase. Synovial cells (SYC) were also harvested from ST and stimulated with vehicle (serum-free culture medium), PGE2, or CGRP.

**Results:**

CGRP, COX-2, and mPGES-1 expression levels were significantly higher in IFPs than STs. PGE2 stimulation increased CGRP expression in Ad, the SVF, and SYC; however, CGRP expression was significantly higher in PGE2-stimulated SVF than PGE2-stimulated SYC. CGRP stimulation had no effect on COX-2 or mPGES-1 expression.

**Conclusions:**

CGRP expression in the IFP of KOA patients is regulated by the COX-2/mPGES-1/PGE2 pathway.

## Background

The infrapatellar fat pad (IFP) is located adjacent to the synovial tissue (ST). Several studies have suggested that factors produced in the IFP are linked to inflammation in the knee joint and could contribute to the development of knee osteoarthritis (KOA) [1, 2]. These factors have not yet been elucidated.

Calcitonin gene-related peptide (CGRP) is a vasodilatory neuropeptide. Several studies have linked elevated levels of CGRP in ST, the IFP, and joint capsule with the development and intensity of osteoarthritis (OA) pain [3–8]. For example, synovial CGRP expression is increased in patients with painful KOA [8]. We previously reported that CGRP is expressed in the IFP. Moreover, CGRP expression levels are correlated with OA grade [9]. These observations suggest that CGRP in the IFP may be a therapeutic target for KOA.

Previous studies have reported that CGRP expression in neural cells, synovial fibroblasts, and epithelial cells is regulated by inflammatory mediators such as tumor necrosis factor-α (TNF-α), interleukin-1β (IL-1β), and prostaglandin E2 (PGE2) [5, 10–12]. IFP also releases inflammatory mediators [13–15]. IFP produced interleukin-6 (IL-6) compared to subcutaneous adipose tissue (ScAT) [14]. Eymard et al. reported that the IFP releases higher levels of PGE2 than subcutaneous adipose tissue (ScAT) [13]. We previously reported a correlation between CGRP expression levels and COX-2 expression in the IFP of KOA patients [9]. CGRP expression in synovial fibroblasts is also regulated by the COX-2/PGE2 pathway.

We hypothesize that PGE2 regulates CGRP expression in the IFP. Here, we investigated CGRP regulation in the IFPs of KOA patients.

## Methods

### Harvesting IFP and ST samples

A sample size of 138 patients was deemed sufficient according to a power calculation using G*POWER3 (alpha = 0.05, power = 0.80). The study subjects included all patients who underwent primary TKA for OA in Kitasato University Hospital. The mean ± SD age of the patients was 73.0 ± 7.8 years, and the mean ± SD body mass index (BMI) was 26.2 ± 4.3 kg/m^2^. The Kellgren-Lawrence (KL) grades among the patients were: KL2 = 3, KL3 = 53, and KL4 = 82 patients. This study protocol was approved by the Kitasato University Medical Ethics Organization (permission number KMEO B13–113). Each patient provided informed consent for participation in this study the day before surgery. IFPs and STs were harvested at the time of total knee replacement surgery. A portion of each IFP and ST sample was instantly frozen in liquid nitrogen and stored at − 80 °C until RNA extraction.

### Real-time polymerase chain reaction

We extracted total RNA and performed cDNA synthesis using ST and IFP samples according to our previously described method [9]. CGRP, COX-2, and mPGES-1 primers were based on previously designed primer sequences [9, 16]. Real-time polymerase chain reaction (RT-PCR) was performed as reported in our previous paper. mRNA expression of our genes of interest was normalized to that of glyceraldehyde dehydrogenase (GAPDH).

### Isolation of synovial cells, the stromal vascular fraction, and adipocytes

To examine the regulation of CGRP expression by PGE2 in IFPs, the stromal vascular fraction (SVF) and adipocytes were extracted from IFPs from eight KOA patients. Adipocytes were isolated from IFPs using a modified version of a previously described method [15]. Briefly, IFP samples were ground up and digested in 1 mg/mL collagenase solution at 37 °C for 2 h with gentle agitation. The solution was filtered and centrifuged at 180 x *g* for 5 min. The floating top layer containing adipocytes was transferred into 25 cm^2^ culture flasks (BD Falcon, Franklin Lakes NJ, USA) filled with Dulbecco’s modified Eagle’s medium (DMEM; Gibco, Carlsbad CA, USA) supplemented with 10% fetal bovine serum (FBS; Tissue Culture Biologicals, Long Beach CA, USA, Lot 106164) and incubated at 37 °C. During the incubation, cells floated up and attached to the upper inner surface of the flask. After 7 days, the medium was decanted and the flasks were inverted so that the adipocytes (Ad) were on the bottom. The floating top layer was collected and the remaining cells were centrifuged at 300 x *g* for 10 min to obtain the SVF. After suspension in culture medium, the SVF was transferred into 25 cm^2^ culture flasks. Synovial cells were also harvested from same the patients as previously described. After culturing for 10 days, Ad, the SVF, and synovial cells were analyzed by RT-PCR to quantify the expression of adipocyte marker genes (adiponectin [Acrp30] and peroxisome proliferator-activated receptor [PPAR-γ]) [17], an endothelial marker (CD31) [18], and mesenchymal stem cell surface markers (CD90, CD105) [19]. Ad, SVF, and synovial cells were stimulated with culture medium with or without 10 μM PGE2 (Cayman Chemical, Ann Arbor MI, USA), and CGRP expression was evaluated by RT-PCR. To evaluate the effect of CGRP on COX-2 and mPGES-1, Ad, SVF, and synovial cells were stimulated with culture medium with or without 100 nM CGRP (Sigma). PGE2 and CGRP concentrations were determined based on a previous study [5, 8, 20].

## Results

CGRP, COX-2, and mPGES-1 mRNA expression in ST and IFPs.

**Fig. 1:**
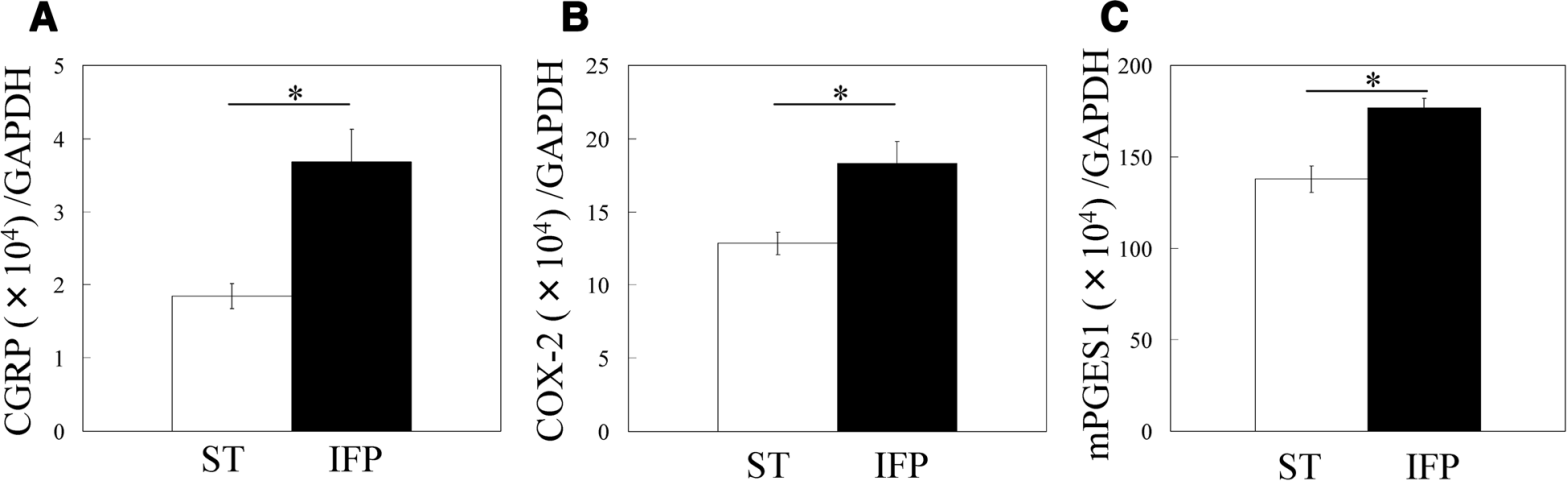
CGRP, COX-2, and mPGES-1 mRNA expression in the infrapatellar fat pad. **a** CGRP, (**b**) COX-2 and (**c**) mPGES-1 mRNA expression in the infrapatellar fat pad (IFP) and synovial tissue (ST) of knee osteoarthritis patients. *Statistically significant difference between IFP and ST (*p* < 0.05). All data are presented as the mean ± standard error (*n* = 138)

CGPR expression was significantly higher in IFPs than ST (2.0-fold, Fig. 1a). IFPs also exhibited significantly higher levels of COX-2 and mPGES-1 compared to ST (1.4-fold and 1.3-fold, respectively, *p* < 0.05; Fig. 1b, c).

### CGRP expression in ad and SVF

**Fig. 2:**
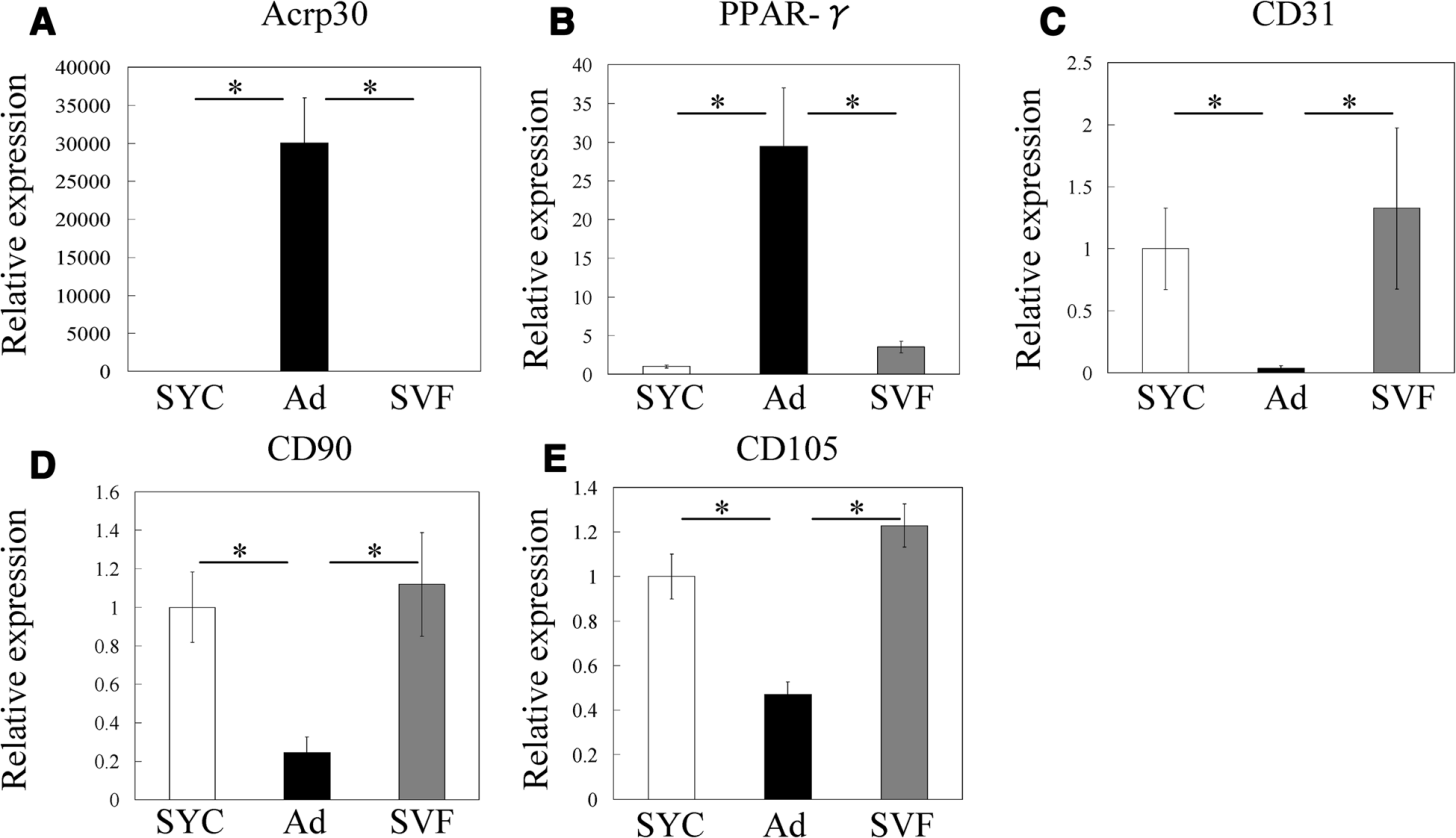
Characterization of cultured synovial cells, adipocytes and the stromal vascular fraction. Expression of the adipocyte marker genes (**a**) Acrp30 and (**b**) PPAR-γ, (**c**) CD31, an endothelial marker, and the fibroblast/stromal cell markers (**d**) CD90 and (**e**) CD105

**Fig. 3:**
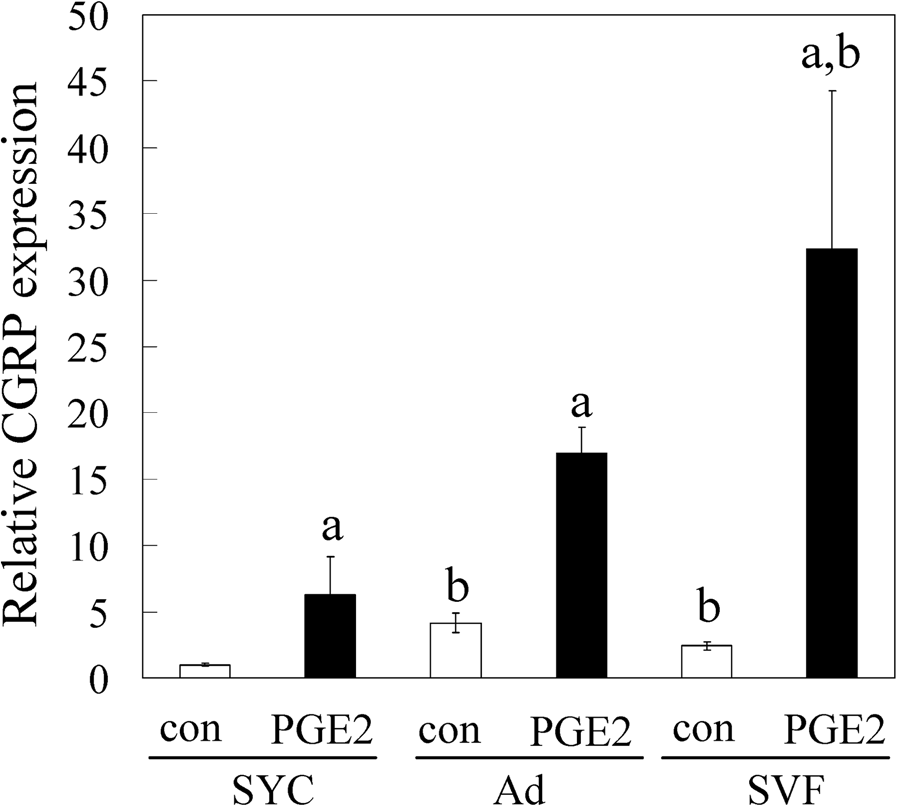
Effects of PGE2 on CGRP expression in cultured synovial cells, adipocytes, and the stromal vascular fraction. Synovial cells (SYC), adipocytes (Ad) and the stromal vascular fraction (SVF) were stimulated in vitro with PGE2 (10 μM) or serum-free medium without PGE2 (control, con) for 8 h. CGRP mRNA expression levels were evaluated by RT-PCR. All data are presented as the mean ± standard error (*n* = 8). *p < 0.05. ^a^ indicates a statistical difference between PGE2-stimulated and non-stimulated cells in each fraction. ^b^ indicates a statistical difference when compared to SYC under the same conditions

RT-PCR showed that Acrp30 and PPAR-γ mRNA expression was significantly higher in Ad than the SVF and synovial cells (Fig. 2a, b). Expression of CD31, CD90, and CD105 was significantly higher in the SVF and synovial cells than in Ad (Fig. 2c–e). These results indicate that we successfully separated the SVF and Ad from IFPs. We subsequently analyzed the effects of PGE2 on CGRP mRNA expression in Ad, SVF, and synovial cells of OA patients by RT-PCR (Fig. 3). CGRP expression in the absence of PGE2 was significantly higher in Ad and the SVF than synovial cells (Fig. 3). Stimulation with PGE2 significantly increased CGRP expression in Ad, SVF, and synovial cells compared to vehicle controls. CGRP expression in PGE2-stimulated SVF was significantly higher than that in PGE2-stimulated synovial cells.

### Effect of CGRP on COX-2 and mPGES-1 expression

**Fig. 4:**
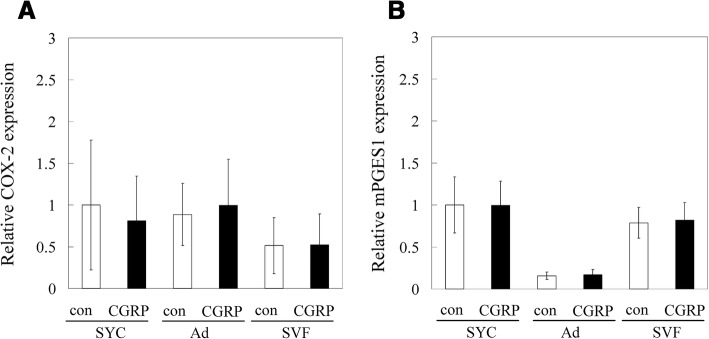
Effects of CGRP on COX-2 and mPGES-1 expression in cultured synovial cells, adipocytes and the stromal vascular fraction. Synovial cells, adipocytes (Ad) and the stromal vascular fraction (SVF) were stimulated in vitro with CGRP (100 nM) or serum-free medium without CGRP (control, con) for 8 h. **a** COX-2 and (**b**) mPGES-1 mRNA expression levels were evaluated by RT-PCR. All data are presented as the mean ± standard error (*n* = 8)

To determine whether CGRP increases COX-2 and mPGES-1 expression, Ad, SVF, and synovial cells were cultured with CGRP. No differences were detected in COX-2 or mPGES-1 expression between CGRP-stimulated and vehicle control cells (Fig. 4a, b).

## Discussion

The expression of CGRP, COX-2, and mPGES-1 was significantly higher in IFPs than ST. PGE2 stimulation increased CGRP expression in adipocytes, SVF, and synovial cells; however, CGRP expression in PGE2-stimulated SVF was significantly higher than that in PGE2-stimulated synovial cells. CGRP stimulation had no effect on COX-2 or mPGES-1 expression. Our results suggest that CGRP expression in the IFP in KOA is regulated by the COX-2/mPGES-1/PGE2 pathway.

Findings from previous studies indicate that CGRP expression in epithelial cells, synovial fibroblasts, macrophages, and T-cells is regulated by inflammatory mediators such as PGE2, TNF-α and IL-1β [5, 8, 10–12, 15, 21, 22]. We previously showed that CGRP expression in ST is correlated with the expression of COX-2 but not TNF-α and IL-1β, and that CGRP expression was increased in synovial fibroblasts following stimulation with PGE2 [5]. In addition, CGRP expression in IFPs is correlated with COX-2 expression [9]. Here, we demonstrated that the IFP expressed higher levels of CGRP and the PGE_2_-forming enzymes COX-2 (conversion of arachidonic acid to PGH2) and mPGES-1 (conversion of COX-2 derived to PGH2 to PGE2) compared to ST. In addition, CGRP expression in adipocytes and the SVF was stimulated by exogenous PGE2. Notably, PGE2-stimulated SVF had higher expression than that in synovial cells. Our findings suggest that COX-2 produced in the IFP, in particular the SVF, may be the source of CGRP in OA, and that CGRP expression in the IFP is regulated by the COX-2/mPGES-1 pathway.

Previous studies have shown that CGRP is expressed in several joint tissues such as the meniscus, joint capsule, ST, and IFP, and suggest that CGRP plays an important role in KOA progression, inflammation, or pain [3–8]. PGE2 is also considered to be a key mediator of inflammatory pain in OA. Non-steroidal anti-inflammatory drugs (NSAIDs), including selective COX-2 inhibitors, constitute the main treatment option for pain in OA [23, 24]. mPGES-1 is expressed during inflammation and, according to mouse gene deletion studies, plays a critical role in inflammatory pain, suggesting that it may be an alternative therapeutic target for pain in KOA [25, 26]. Together, our findings and previous studies suggest that the COX-2/mPGES-1/PGE2/CGRP pathway may have an important role in OA pathophysiology.

There are two limitations of the present study. First, we did not examine a non-KOA patient group. Future studies should examine whether CGRP levels are higher in the IFPs of OA patients compared to normal subjects. Second, although CGRP levels were elevated in IFPs compared to that in STs, the relationship between CGRP and KOA pathology remains to be determined.

## Conclusion

CGRP expression in the IFP of KOA patients is regulated by the COX-2/mPGES-1/PGE2 pathway. Further investigations may reveal more details of the pathophysiology and suggest additional therapeutic targets for KOA.

## Abbreviations

Acrp30: Adiponectin
CGRP: Calcitonin gene-related peptide
COX-2: Cyclooxygenase-2
IFP: Infrapatellar fat pad
IL-1β: Interleukin-1β
IL-6: Interleukin-6
KOA: Knee osteoarthritis
mPGES-1: Microsomal prostaglandin E synthase-1
NSAIDs: Non-steroidal anti-inflammatory drugs
PCR: Polymerase chain reaction
PGE-2: Prostaglandin E2
PPAR-γ: Peroxisome proliferator-activated receptor
ScAT: Subcutaneous adipose tissue
ST: Synovial tissues
SVF: Stromal vascular fraction
TKR: Total knee replacement
TNF-α: Tumor necrosis factor-α
